# Comparison of DWI and ^18^F-FDG PET/CT for assessing preoperative N-staging in gastric cancer: evidence from a meta-analysis

**DOI:** 10.18632/oncotarget.21055

**Published:** 2017-09-19

**Authors:** Mingxu Luo, Hongmei Song, Gang Liu, Yikai Lin, Lintao Luo, Xin Zhou, Bo Chen

**Affiliations:** ^1^ Department of Gastrointestinal Surgery, Xiamen Cancer Hospital, The First Affiliated Hospital of Xiamen University, Xiamen, China; ^2^ Department of Oncology, Renmin Hospital of Shiyan, Hubei University of Medicine, Shiyan, China; ^3^ Department of Radiology, The First Affiliated Hospital of Xiamen University, Xiamen, China; ^4^ Teaching and Research Section of Surgery, The First Clinical College of Fujian Medical University, Fuzhou, China

**Keywords:** lymph node staging, gastric cancer, diffusion weighted imaging, positron emission tomography/computed tomography

## Abstract

The diagnostic values of diffusion weighted imaging (DWI) and ^18^F-fluorodeoxyglucose positron emission tomography/computed tomography (^18^F-FDG PET/CT) for N-staging of gastric cancer (GC) were identified and compared. After a systematic search to identify relevant articles, meta-analysis was used to summarize the sensitivities, specificities, and areas under curves (AUCs) for DWI and PET/CT. To better understand the diagnostic utility of DWI and PET/CT for N-staging, the performance of multi-detector computed tomography (MDCT) was used as a reference. Fifteen studies were analyzed. The pooled sensitivity, specificity, and AUC with 95% confidence intervals of DWI were 0.79 (0.73–0.85), 0.69 (0.61–0.77), and 0.81 (0.77–0.84), respectively. For PET/CT, the corresponding values were 0.52 (0.39–0.64), 0.88 (0.61–0.97), and 0.66 (0.62–0.70), respectively. Comparison of the two techniques revealed DWI had higher sensitivity and AUC, but no difference in specificity. DWI exhibited higher sensitivity but lower specificity than MDCT, and ^18^F-FDG PET/CT had lower sensitivity and equivalent specificity. Overall, DWI performed better than ^18^F-FDG PET/CT for preoperative N-staging in GC. When the efficacy of MDCT was taken as a reference, DWI represented a complementary imaging technique, while ^18^F-FDG PET/CT had limited utility for preoperative N-staging.

## INTRODUCTION

Although the incidence and mortality have dramatically decreased over the past 50 years, gastric cancer (GC) remains the fourth common cancer and the second leading cause of cancer-related deaths, with poor prognosis worldwide [1, 2]. The variety of therapeutic options available for GC, such as radical resection, endoscopic submucosal dissection, and neoadjuvant chemotherapy [3], makes accurate preoperative TNM staging for GC patients a necessity [4–6]. Lymph node assessment is crucial to treatment strategy and to determining prognosis in GC patients [7, 8]. In cases without distant metastases, extended lymphadenectomy based on precise lymph node staging is an important procedure in radical gastrectomy, which could improve the outcome for GC patients [9, 10]. According to Japanese Gastric Cancer Association, for differentiated T1a early GC without lymph node metastasis, endoscopic resection or partial resection plus D1/D1+ lymphadenectomy is indicated, but patients with lymph node metastasis need a standard D2 lymphadenectomy [11]. Closely correlated with tumor size, infiltrating degree, and vascular tumor thrombus, lymph node metastasis is regarded as a key independent predictor of recurrence and is one of the indications for adjuvant chemotherapy in GC patients [10, 12]. Statistically, the 5-year survival rate (after surgical treatment) in patients with N0 GC is 86.1%, whereas the survival rates in patients with N1, N2, and N3 GC dramatically decrease to 58.1%, 23.3%, and 5.9%, respectively [13]. Therefore, accurate preoperative lymph node assessment might facilitate the selection of candidates for neoadjuvant chemotherapy, optimize radical surgery strategy, and predict prognosis of GC [14].

Several tools to diagnose lymph node metastasis of GC are available, such as multi-detector computed tomography (MDCT), endoscopic ultrasonography (EUS), positron emission tomography/computed tomography (PET/CT), and magnetic resonance imaging (MRI) [15]. MDCT is most widely used to assess lymph node staging of GC patients, mainly on the basis of lymph node size [16, 17], but the limited sensitivity of MDCT results in false negative findings [18–21]. EUS provides good information on lymph node status around lesions but was inadequate for predicting extra-perigastric and distant lymph node metastasis because of the limited penetration range of the ultrasound beam [15, 22]. Therefore, finding more accurate imaging techniques for N-staging of GC is essential.

Diffusion weighted imaging (DWI) and ^18^F-Fluorodeoxyglucose positron emission tomography/computed tomography (^18^F-FDG PET/CT) are relatively new imaging techniques used for preoperative staging of numerous cancers. Studies have suggested that diffusion MRI is helpful in distinguishing malignant from benign lesions by use of apparent diffusion coefficient (ADC) measurements [23–27]. The theory is that malignant tumors have restricted diffusion whereas benign lesions do not [28, 29]. Although the value of this imaging modality in the differentiation of metastatic lymph nodes from non-metastatic lymph nodes has been shown in patients with neck, lung, prostate and colorectal cancers [30–33], no enough evidence is available to support the generally accepted use of DWI in nodal staging of GC patients.

^18^F-FDG PET/CT, which integrates the anatomical details from CT with the functional status from PET, facilitates early detection of primary lesions and differentiation of metastases in various cancers, including GC [34]. PET/CT have several advantages to PET alone or CT alone, and PET/CT is increasingly used in diagnostic staging, treatment decisions and prognosis evaluations [35–37]. The usefulness of PET/CT in the assessment of preoperative lymph node involvement is hindered by unsatisfactory sensitivity compared with contrast-enhanced CT, despite PET/CT showing better specificity [38, 39]. Furthermore, the few published studies on the subject exhibited a wide range of sensitivities and specificities in the preoperative diagnostic performance of ^18^F-FDG-PET/CT in nodal assessment of GC [40, 41].

The value of conventional imaging techniques, such as MDCT, EUS, MRI, and PET, has been investigated by meta-analyses [42–45]. However, the efficacy of DWI and ^18^F-FDG-PET/CT in lymph node staging were not determined and no relevant meta-analyses were performed. Therefore, we performed a systematic review and meta-analysis to confirm and compare the diagnostic values of DWI and ^18^F-FDG PET/CT for lymph node staging in GC patients.

## RESULTS

### Study selection and description

**Figure 1:**
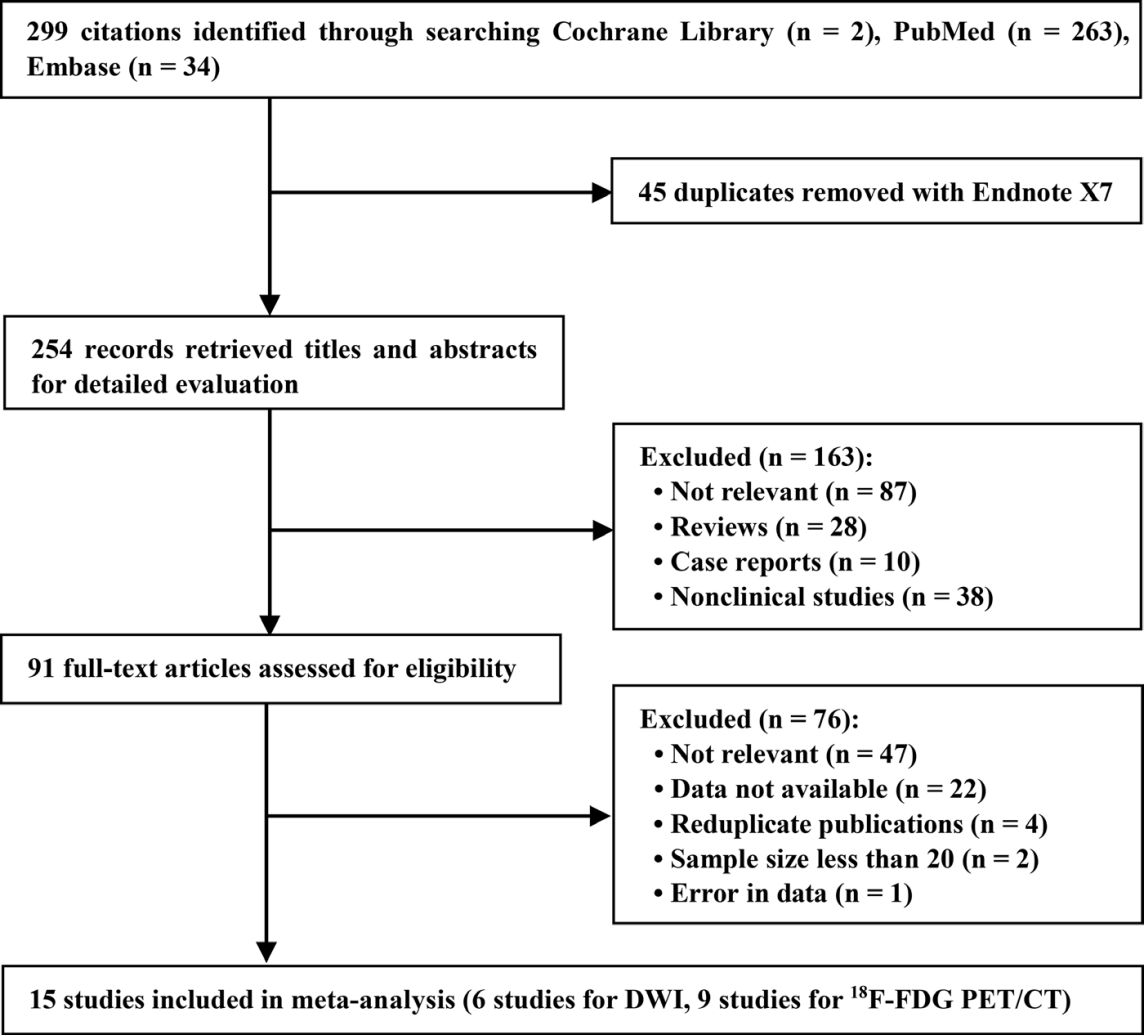
Flow diagram of literature search and study selection

A total of 299 articles were screened in the primary literature search. After removing the ineligible study in each step, 15 studies (six studies [46–51] for DWI and nine studies [21, 38–41, 52–55] for ^18^F-FDG PET/CT were finally selected on basis of the inclusion and exclusion criteria. A flowchart depicting the study selection process is shown in Figure 1.

The principal characteristics of the 15 selected articles are listed in Table 1. Of these articles, 12 were retrospective, and three were prospective. Patients in 11 articles were Asians while another four articles were Caucasians. All the reference standards are based on pathological analysis after surgery, although the operation methods differed. Considering the complexity of the MRI technique, Table 2 summarizes the field strength, imaging evaluation, b value, the number of reporting radiologists, pulse sequence and diagnostic criteria of DWI in each study. Similarly, the characteristics of ^18^F-FDG PET/CT in nine studies are displayed in Table 3.

**Table 1:** Principle characteristics of included studies

Author (Year)	Country (Ethnicity)	Number	Design	Mean age (Years)	Gender (M/F)	Imaging examination	Reference standard*
Giganti 2017 [46]	Italy (Caucasian)	89	R	71 (66–78)^#^	58/31	DWI	Pathological analysis after Ivor-Lewis (*n* = 5), subtotal gastrectomy (*n* =52) and total gastrectomy (*n* =32)
Giganti 2016 [47]	Italy (Caucasian)	52	P	68.5 (43-85)	33/19	DWI	Pathological analysis after Ivor-Lewis (*n* = 2), subtotal gastrectomy (*n* =28) and total gastrectomy (*n* =22)
Joo 2015 [48]	Korea (Asian)	47	P	61.5 (38–91)	ND	DWI	Pathological analysis after curative or palliative gastrectomy and LN dissection
Hasbahceci 2015 [49]	Turkey (Caucasian)	23	P	59.4 ± 10.9^$^	11/12	DWI	Pathological analysis after radical resection of gastric tumor with standard D1+ or D2 LN dissection
Zhou 2014 [50]	China (Asian)	52	R	60 (28–80)	34/18	DWI	Pathological analysis after D1 (*n* = 15), D2 (*n* =24) and D3 lymphadenectomy (*n* = 13)
Lei 2013 [51]	China (Asian)	39	R	52 (31–82)	26/12	DWI	Pathological analysis after surgery
Altini 2015 [52]	Italy (Caucasian)	45	R	66 (44–86)	27/18	PET/CT	Pathological analysis after surgery
Filik 2015 [41]	Turkey (Caucasian)	31	R	58.9±12.6	24/7	PET/CT	Pathological analysis after curative surgery including gastrectomy and lymph node dissection
Namikawa 2014 [53]	Japan (Asian)	90	R	72 (19–89)	70/20	PET/CT	Pathological analysis after gastrectomy: D2 (*n* = 56), D1 (*n* =25), D0 (*n* = 9)
Park 2014 [39]	Korea (Asian)	74	R	67 (38–88)	56/18	PET/CT	Pathological analysis after standard gastrectomy and regional LN dissection (at least D2 dissection)
Youn 2012 [40]	Korea (Asian)	396	R	59 (27–86)	278/118	PET/CT	Pathological analysis after radical subtotal or total gastrectomies (*n* = 384), open and closure (*n* = 4) and palliative surgery (*n* = 8)
Ha 2011 [21]	Korea (Asian)	78	R	61 (31–85)	53/25	PET/CT	Pathological analysis after standard lymphadenectomy (at least D2).
Kim 2011 [38]	Korea (Asian)	71	R	58 (27–77)	53/25	PET/CT	Pathological analysis after radical surgery such as total gastrectomy *n* = 30) or subtotal gastrectomy (*n* =41) in conjunction withlymphadenectomy
Oh 2011 [54]	Japan (Asian)	136	R	64.4 ± 10.5	98/38	PET/CT	Pathological analysis after gastrectomy
Yang 2008 [55]	Japan (Asian)	78	R	65.6 ± 1.1	57/21	PET/CT	Pathological analysis after radical gastrectomy (D1+beta for EGC, D2 for AGC)

^#^Data in brackets were represented as age range; ^$^Data were represented as mean ± standard deviation. *D0, D1, D1+, D2 and D3 refers to the classification of LN dissection depending on the extent of lymph nodes removed at the time of gastrectomy. Abbreviations: DWI = diffusion weighted imaging; PET/CT = positron emission tomography/computed Tomography; P = prospective; R = retrospective; EGC = early gastric cancer; AGC = advanced gastric cancer; L*N* =lymph node; M = male; F = female; ND = not documented.

**Table 2:** Characteristics of DWI of included studies

Study, year	Field strength	Imaging evaluation	B value (s/mm^2^)	Number of reporting radiologists	Pulse sequences	Diagnostic criteria
Giganti, 2017	1.5T	QL and QN	0, 600	Two radiologists (independently)	Multiplanar T2-weighted study, followed by a DW-MRI protocol and a dynamic T1-weighted study	Quantitative measurements were obtained tracing a small region of interest on the ADC map, so as to minimize partial volume effects
Giganti, 2016	1.5T	QL	0, 600	Two radiologists (independently)	Dynamic T1WI with fat suppression, T2WI with and without fat suppression, DWI	SAD ≥ 6 mm for round perigastric LNs and hyperintensity on DWI
Hasbahceci,2015	1.5T	QL and QN	50, 400 and 800	One radiologist	Axial and coronal TSE-T1WI, axial and coronal fat saturated TSE-T2WI, axial and coronal SPGR-T1WI, axial T2W fat saturated sequence, axial SS-SE-EP DWI with a selective fat suppression	QL: SAD ≥ 5 mm with heterogeneous enhancement, or heterogeneous signal intensity than muscle as seen on DWI. QN: ADC value < 1.1 × 10^-3^ mm^2^/s
Joo, 2015	3T	QL	0, 100, 500, and 1000	Two radiologists (in consensus)	Axial GRE-3D-T1W, HASTE-T2W, True-FISP, DWI	SAD ≥ 8 mm or any LN with higher signal intensity than muscle on DWI with b values of 500 or 1000^2^/sec
Zhou, 2014	3T	QN	0, 1000	Two radiologists (independently)	TSE-T2WI without fat suppression, SS-SE-EP DWI with fat suppression.	ADC value < 1.189 × 10^-3^mm^2^/s
Lei, 2013	1.5T	QL	600	ND	Cross-sectional and coronal oblique T1 FSPGR, T2 SSFSE, T2 ASSET, DWI	SAD of perigastric LN > 5 mm and distalis perigastric LN > 6 mm

Abbreviations: QL = qualitative; Q*N* = quantitative; SAD = short-axis diameter; L*N* =lymph node; MRI = magnetic resonance imaging; DWI = diffusion weighted imaging; T1WI = T1 weighted imaging; T2WI = T2 weighted imaging; ADC = apparent diffusion coefficient; SE = Spin echo; TSE = turbo spin-echo; SPGR = spoiled gradient recalled echo; SS-SE-EP = single-shot spin-echo echo-planar imaging; GRE = gradient recalled echo; HASTE = half-fourier acquisition single-shot turbo spin-echo; True-FISP = true fast imaging with steady-state precession; FLASH = fast low angle shot; SSFSE = single-shot fast spin-echo; ASSET = array spatial sensitivity encoding technique; FSPGR = fast spoiled gradient-recalled; ND = not documented.

**Table 3:** Characteristics of ^18^F-FDG PET/CT of included studies

Study (year)	Manufacturer	CT Scanner (detector rows, slice thickness)	Imaging evaluation	Injected dose	Number of reviewers	Diagnostic criteria of positive lymph node metastases
Altini 2015	GE	16, 3.75 mm	QL	4.6 MBq/kg^#^	a nuclear physician	Higher ^18^F-FDG uptake in at least one lymph node
Filik 2015	GE	ND, 5 mm	QL	8-10 mCi	ND	Higher ^18^F-FDG uptake than adjacent tissues and blood pool activity
Namikawa 2014	GE	14, 1.25 mm	QL	3.5 MBq/kg	ND	^18^F-FDG uptake similar to or higher than that of the liver
Park 2014	GE	8, 1.25 mm	QL	7.4 MBq/kg	ND	^18^F-FDG uptake similar to or higher than that of the blood pool
Youn 2012	Siemens	ND, 5 mm	QL	ND	One nuclear physician	Higher ^18^F-FDG uptake than normal tissues
Ha 2011	Siemens	ND, 5 mm	QL	5-6 MBq/kg	ND	^18^F-FDG uptake of lymph node bearing areas regardless of size
Kim 2011	GE	ND, 4.3 mm	QL	370 MBq	Two subspecialty-trained abdominal radiologists and one nuclear medicine physician (in consensus)	A focal ^18^F-FDG uptake was higher than the normal biodistribution of background FDG activity
Oh 2011	Philips	ND, ND	QN	7.4 MBq/kg	ND	P-SUV > 3.2 kBq/mL or higher ^18^F-FDG uptake in lymph nodes
Yang 2008	GE	ND, ND	QL	200 MBq	ND	Higher ^18^F-FDG uptake in at least one lymph node

^#^MBq/kg meant that the injected dose of ^18^F-fluorodeoxyglucose was based on the weight of patients who received PET/CT scanning. Abbreviations: CT: computed tomography; GE = American General Corporation; ^18^F-FDG = ^18^F-fluorodeoxyglucose; QL = qualitative analysis; Q*N* = quantitative analysis; ND = not documented; P-SUV = peak-standardized uptake value.

### Quality assessment

**Figure 2:**
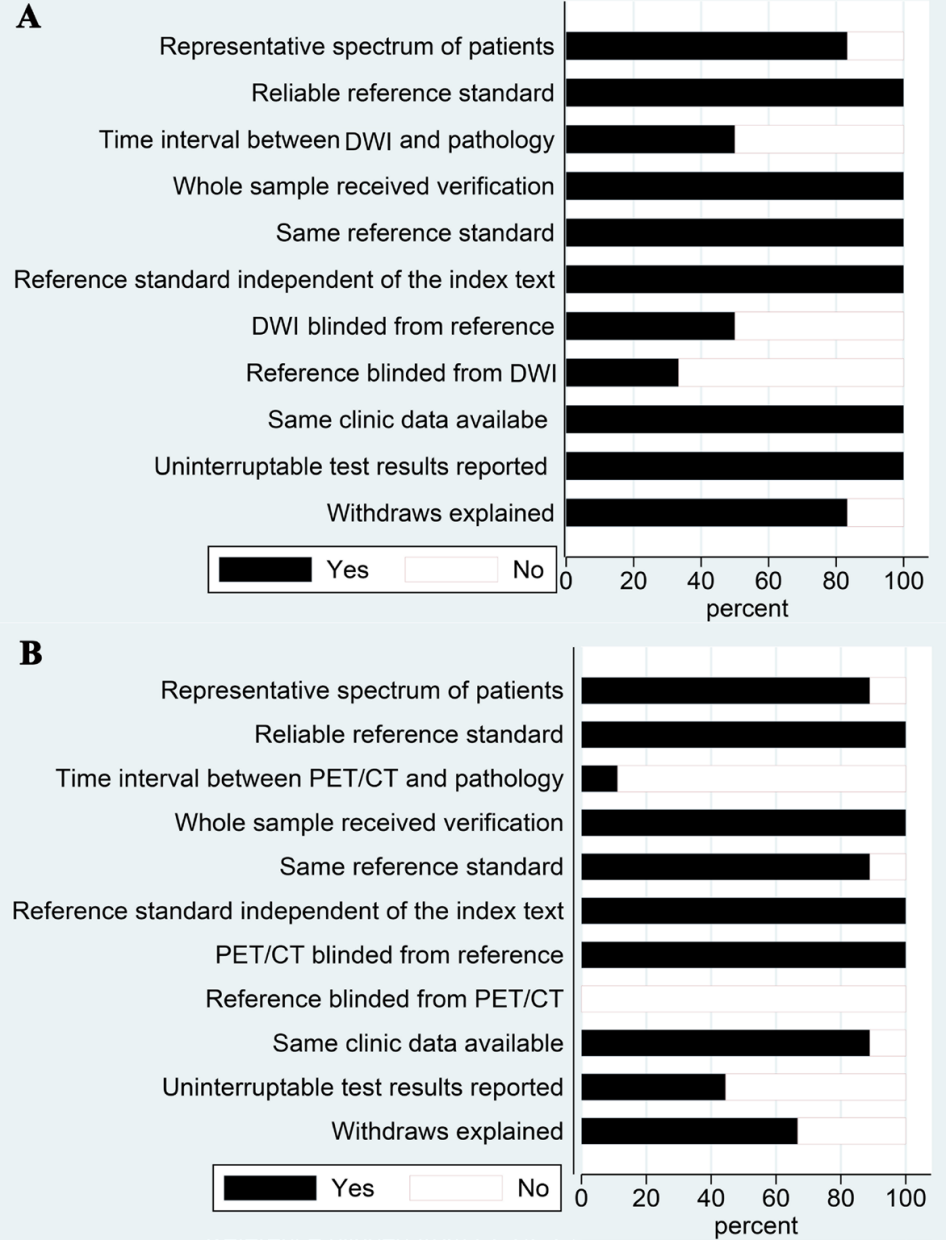
Quality assessment of included studies using QUADAS-2 (**A**) For DWI studies; (**B**) For ^18^F-FDG PET/CT studies.

Figure 2 showed the methodological quality assessment for six studies of DWI and nine studies of ^18^F-FDG PET/CT. All the included studies used pathological diagnosis as a reference. There of six DWI studies and only one of nine ^18^F-FDG PET/CT studies reported time intervals between examinations and pathological confirmations. Six of six DWI studies and eight of nine ^18^F-FDG PET/CT studies had the same reference standard. Two of six DWI studies reported that references were blinded from MRI and no studies described blind measurements of reference tests without knowledge of ^18^F-FDG PET/CT. Six of six DWI studies and six of nine ^18^F-FDG PET/CT studies provided clinical data when interpreting the two imaging techniques.

### Diagnostic accuracy of DWI and ^18^F-FDG PET/CT

**Figure 3:**
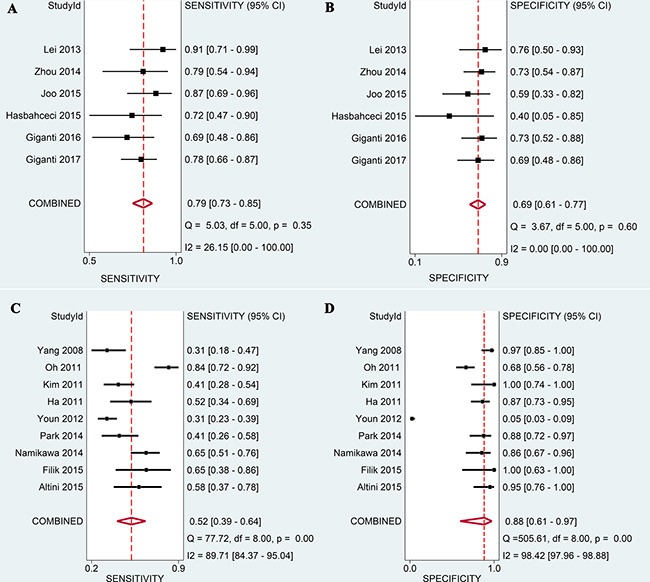
Forest plots of DWI and ^18^F-FDG PET/CT in evaluating preoperative N-staging in patients with gastric cancer (**A**) Pooled sensitivity of DWI; (**B**) Pooled specificity of DWI; (**C**) Pooled sensitivity of ^18^F-FDG PET/CT; (**D**) Pooled specificity of ^18^F-FDG PET/CT.

**Figure 4:**
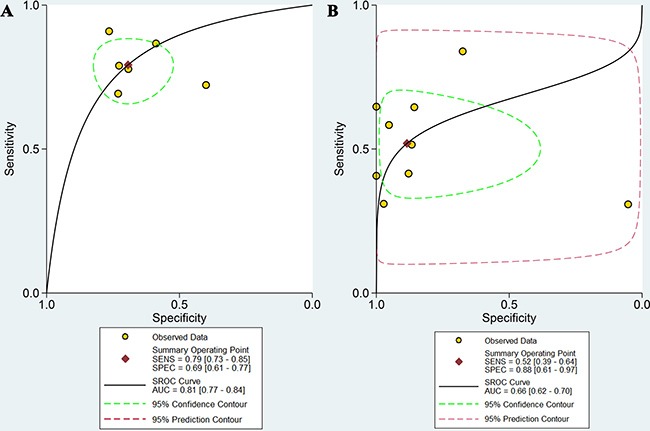
The summary ROC curves of DWI and ^18^F-FDG PET/CT in evaluating preoperative N-staging in patients with gastric cancer (**A**) For DWI; (**B**) For ^18^F-FDG PET/CT .

The pooled results are shown in Figure 3 and Table 4. On the basis of six studies, DWI had a sensitivity of 0.79 (95% CI: 0.73–0.85) and a specificity of 0.69 (95% CI: 0.61–0.77). In nine studies, PET/CT achieved a sensitivity and specificity of 0.52 (95% CI: 0.39–0.64) and 0.88 (95% CI: 0.61–0.97), respectively. The fitted summary receiving operator characteristics (sROC) curves estimated the area under summary receiver operating characteristic curves (AUCs) of 0.81 (95% CI: 0.77–0.84) for DWI and 0.66 (95% CI: 0.62–0.70) for ^18^F-FDG PET/CT (Figure 4).

**Table 4:** Comparison of diagnostic efficacy of preoperative N-staging in gastric cancer using ^18^F-FDG PET/CT, DWI and MDCT based on meta-analyses

Study	Techniques	Sensitivity (95% CI)	Specificity (95% CI)	AUC (95% CI)
Present study	PET/CT	0.52 (0.39–0.64)	0.88 (0.61–0.97)	0.66 (0.62–0.70)
Present study	DWI	0.79 (0.73–0.85)	0.69 (0.61–0.77)	0.81 (0.77–0.84)
Wang et al. [43]	MDCT	0.67 (0.66–0.69)	0.84 (0.83–0.85)	0.83 (ND)

Abbreviations: ^18^F-FDG PET/CT, ^18^F-fluorodeoxyglucose positron emission tomography/computer tomography; DWI = diffusion weighted imaging; MDCT = multi-detector computed tomography; 95% CI = 95% confidence intervals; AUC = area under summary receiver operating characteristic curve; ND = not documented.

To confirm the summary estimates of two imaging techniques in the evaluation of nodal staging of GC patients, we conducted the comparison between DWI and ^18^F-FDG PET/CT on the pooled sensitivity, specificity and AUC by using the Z test. The results indicated that DWI had an advantage over ^18^F-FDG PET/CT in sensitivity (0.79 vs. 0.52, *P* < 0.001) and AUC (0.81 vs. 0.66, *P* < 0.001), and no differences in specificity between the two imaging examinations was detected (0.69 vs. 0.88, *P* = 0.06).

To better understand the clinical diagnostic performance of the two imaging techniques, we used the corresponding values of MDCT from Wang's meta-analysis as a reference, which was published in 2015 [43]. This meta-analysis included 30 studies, and pooled the sensitivity, specificity and AUC were 0.67, 0.84 and 0.83, respectively (Table 4). When compared with MDCT, DWI had higher sensitivity (0.79 vs. 0.67, *P* < 0.001) but lower specificity (0.69 vs. 0.84, *P* < 0.001), and ^18^F-FDG PET/CT had lower sensitivity (0.52 vs. 0.67, *P* < 0.001) and equivalent specificity (0.88 vs. 0.84, *P* = 0.66). For value of AUC, neither the DWI (0.81) nor ^18^F-FDG PET/CT (0.66) had advantages over MDCT (0.83) in preoperative lymph node assessment of GC.

### Heterogeneity analysis

Our analysis disclosed strong heterogeneity in both sensitivity (I^2^ = 89.7%, *P* < 0.001) and specificity (I^2^ = 98.4%, *P* < 0.001) among ^18^F-FDG PET/CT studies instead of DWI (I^2^ = 26.2%, *P* = 0.35 for sensitivity, I^2^ = 0.0%, *P* = 0.60 for specificity). The Spearman rank correlation test indicated the absence of a threshold effect both in DWI studies (coefficient = 0.27, *P* = 0.52) and in ^18^F-FDG PET/CT studies (coefficient = 0.30, *P* = 0.62). To further identify the resources of heterogeneity for ^18^F-FDG PET/CT studies, meta-regression and subgroup analyses were performed on the basis of the ethnicity of subjects, number of subjects in each included study (sample size larger than 100 vs. sample size smaller than 100), the manufacturer of PET/CT (General Electric [GE] vs. non-GE) and imaging evaluation (qualitative analysis vs. quantitative analysis).

**Figure 5:**
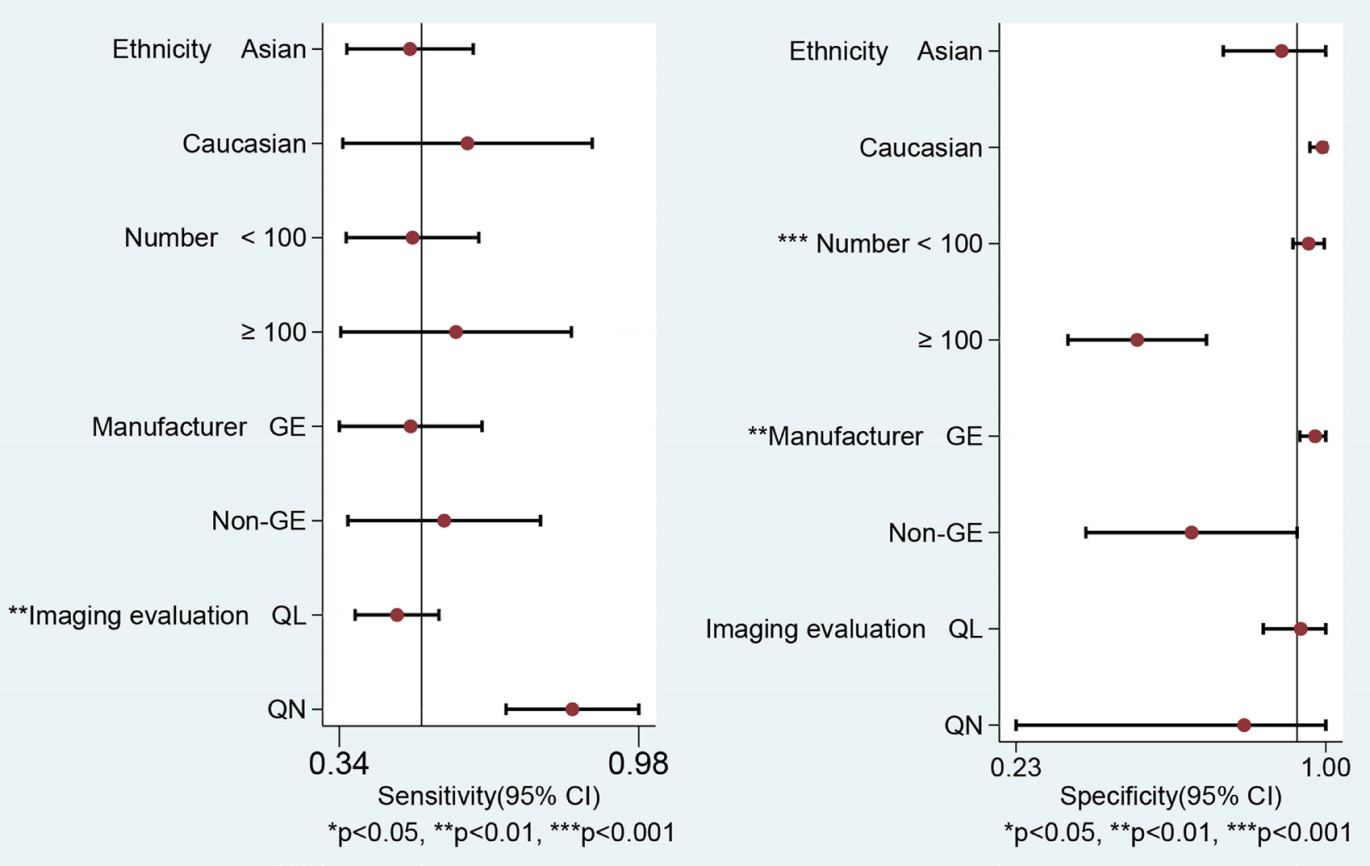
Univariable meta-regression & subgroups analyses of diagnostic performance of ^18^F-FDG PET/CT (Abbreviations: GE = American General Corporation; QL = qualitative analysis; QN *=* quantitative analysis).

**Figure 6:**
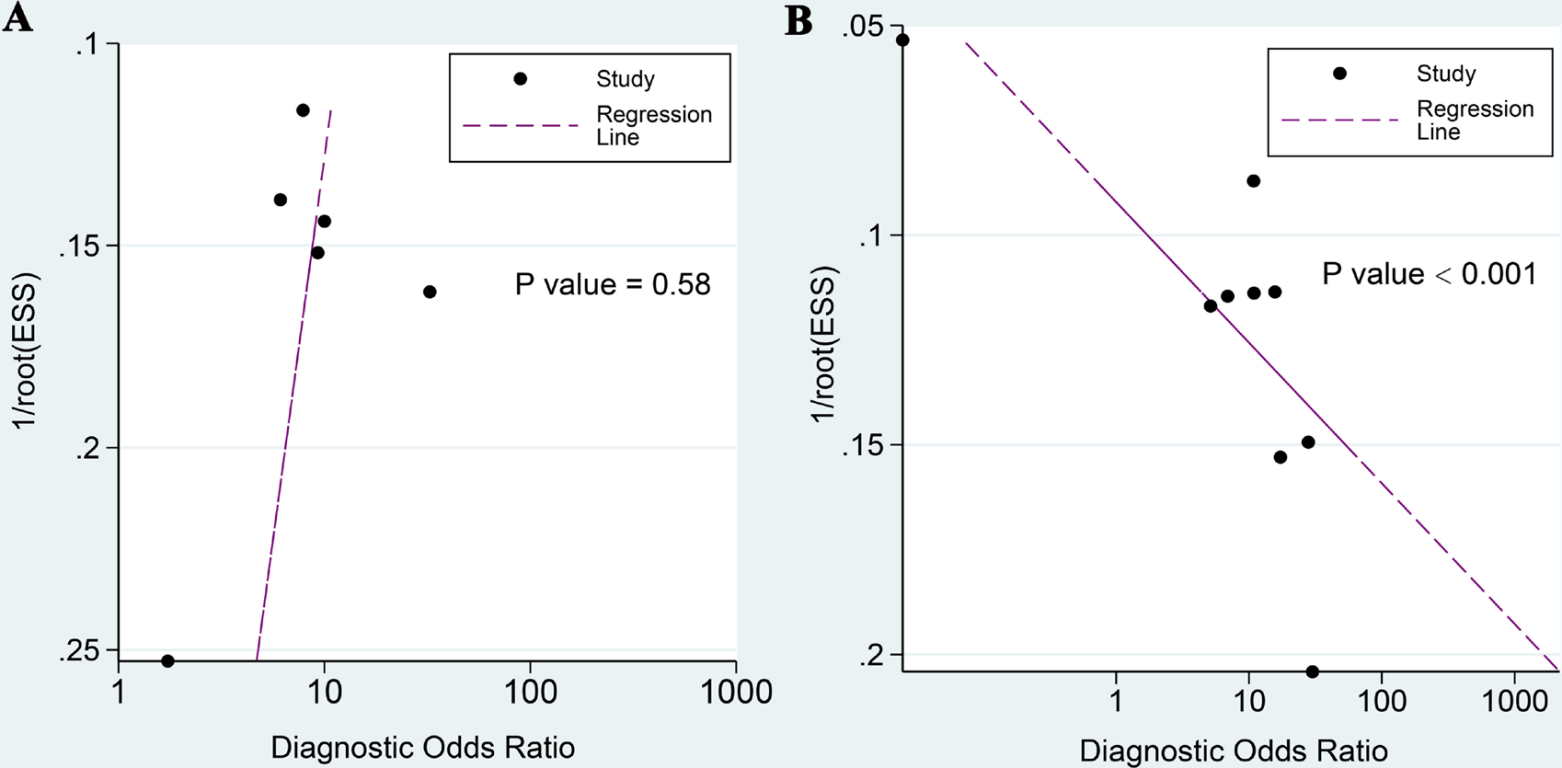
Deeks’ funnel plot asymmetry tests for assessing potential publication bias (**A**) For DWI; (**B**) For ^18^F-FDG PET/CT.

The univariable meta-regression and subgroups analyses of sensitivity and specificity of ^18^F-FDG PET/CT are presented in Figure 5 and Table 5. Eight studies that utilized qualitative analyses showed much lower sensitivity than in quantitative analyses (0.47 vs. 0.84, *P* < 0.001) but failed to explain the heterogeneity of specificity. Six studies that utilized GE equipment exhibited a higher specificity (0.96 vs. 0.47, *P* < 0.001) than a study that utilized non-GE equipment. Seven studies with the number of subjects < 100 showed higher specificity than studies with the number of subjects > 100 (0.93 vs. 0.25, *P* < 0.001). The ethnicity of participants failed to explain the heterogeneity (*P* = 0.44 for sensitivity, *P* = 0.83 for specificity, respectively). Deeks’ funnel plots provided evidence of publication bias for PET/CT studies (*P* < 0.001) rather than DWI studies (*P* = 0.58) (Figure 6).

**Table 5:** The results of subgroup analysis for ^18^F-FDG PET/CT

subgroups	No. of studies	Sensitivity (95% CI)	*P* Value	Specificity (95%)	*P* Value
**Ethnicity**					
Asian	6	0.49 (0.36–0.63)	0.44	0.83 (0.59–1.00)	0.63
Caucasian	3	0.62 (0.35–0.88)		0.99 (0.94–1.00)	
**Number of subjects**					
< 100	7	0.50 (0.36–0.64)	0.53	0.93 (0.87–0.99)	0.00
≥ 100	2	0.59 (0.35–0.84)		0.25 (0.02–0.53)	
**Manufacturer**					
GE	6	0.50 (0.34–0.65)	0.60	0.96 (0.90–1.00)	0.00
Non-GE	3	0.57 (0.36–0.77)		0.47 (0.05–0.89)	
**Imaging evaluation**					
Qualitative analysis	8	0.47 (0.38–0.56)	0.00	0.90 (0.75–1.00)	0.06
Quantitative analysis	1	0.84 (0.72–0.92)		0.68 (0.23–1.00)	

Abbreviations: GE = American General Corporation; 95% CI = 95% confidence intervals.

## DISCUSSION

The treatment strategies and prognoses of GC subjects are heavily dependent on accurate staging before surgery. Generally, preoperative N-staging assessment based on imaging modalities, compared with T-staging, remains less precise and leaves much room for improvement [56–58]. Among the conventional imaging modalities for lymph node evaluation of GC patients, the value of MDCT, EUS, MRI and PET have been investigated by meta-analyses [42–44]. DWI and PET/CT are updated imaging techniques, but their diagnostic efficacy for lymph node involvement in GC has been inconsistently reported [40, 41, 49, 51]. We performed this systematic review and meta-analysis to provide evidence for a better selection for imaging assessment of metastatic lymph node in patients with GC.

Among the 15 DWI and ^18^F-FDG PET/CT studies included in our meta-analysis, DWI achieved a higher sensitivity than PET/CT for lymph node staging in GC patients (0.79 vs. 0.52, respectively, *P* < 0.001). However, no difference in specificity between the DWI and ^18^F-FDG PET/CT was detected (0.69 vs. 0.88, respectively, *P* = 0.06). Consequently, the superiority of DWI can be explained by the observation that DWI produced fewer false-negative results (1 – sensitivity) for N staging of GC. However, the specificity was not fully satisfactory, and thus excessive treatment and excision range might occur because of a relatively greater false-positive results (1 – specificity). The poor sensitivity of ^18^F-FDG PET/CT resulted in a high number of false-negative findings (1–sensitivity), which was similar to the results of Yun et al. [59] and Yang et al. [55], suggesting that positive lymph nodes would be missed and potentially resectable GC patients would receive inappropriate therapy.

The sROC curve and its AUC are used to describe the relation between the sensitivity and specificity in a study and the overall estimation of test performance [60]. A preferred test has an AUC close to 1, whereas a poor test has an AUC close to 0.5. The AUC for DWI is significantly higher than that for ^18^F-FDG PET/CT (0.81 vs. 0.66, *P* < 0.001), indicating that DWI might be more accurate for nodal staging in GC patients. However, neither of the AUCs of the two techniques are high enough to be sufficient for nodal staging of GC patients in clinical practice.

Currently, MDCT is the most frequently used imaging modality for GC staging before surgery [61]. To better understand the clinical value of DWI and ^18^F-FDG PET/CT for N-staging of GC patients, we compared the summarized sensitivities, specificities, and AUCs of the two imaging modalities with those of MDCT in a previous meta-analysis performing by Wang et al. [43]. This meta-analysis covering 6,726 subjects estimated the sensitivity, specificity, and AUC to be 0.67, 0.84, and 0.83, respectively. The poor sensitivity of MDCT is not adequate for the detection of metastasized lymph nodes, so it is essential to obtain the accuracy of other imaging techniques for N-staging of GC patients and analyze the possibilities of these techniques replacing MDCT. In this study, DWI achieved higher sensitivity but lower specificity, and ^18^F-FDG PET/CT had lower sensitivity and equivalent specificity when compared with MDCT (data are shown in Table 4). DWI and ^18^F-FDG PET/CT had no obvious advantages of AUC over MDCT in preoperative lymph node assessment of GC patients, and the two techniques are more costly and require longer scanning times than MDCT. Thus, DWI and ^18^F-FDG PET/CT were unlikely to take the place of MDCT in the short term for lymph node staging of GC patients. Nevertheless, the higher sensitivity and lower specificity of DWI indicates that DWI and MDCT could be complementary imaging modalities and the combined utilization of the two techniques might improve the accuracy of lymph node staging [62].

DWI, a magnetic resonance imaging (MRI) technique, can recognize the restricted diffusion of water molecules among tissues at the cellular level by the measurement of ADC value [23, 63]. DWI has increasingly been used to characterize various diseases and diseased lymph nodes, including alimentary tract cancers such as gastric or colorectal cancers, and has shown promising results [27, 64–66]. However, the value of DWI in the detection and characterization of lymph nodes in GC remains controversial [48, 49, 64]. In the past, DWI of the abdomen and pelvis was easily distorted by respiratory motion and gastrointestinal peristalsis [67, 68]. Recent technological developments in MRI, including new sequences (echo-planar imaging sequence, multichannel coils and parallel imaging), the high-field magnet and volumetric acquisition of T1-weighted images, allow the acquisition of DWI that is largely free of motion artifacts and provide excellent anatomical detail [23, 69, 70]. By performing this meta-analysis, we found that DWI displays an acceptable sensitivity and moderate specificity for N-staging, but based on the AUC value, the DWI is not adequate for nodal staging of GC patients in clinical practice.

In the N-staging of GC patients, the accuracy of DWI is poor when based only on the size of lymph node in imaging, but when integrated with the ADC value as the diagnostic standard, the detection rate is much improved [23, 50, 71]. Zhou et al. [50] reported that the mean ADC value of metastatic lymph nodes (1.059 × 10^–3^ mm^2^/s) was lower than that of non-metastatic lymph nodes (1.4029 × 10^–3^ mm^2^/s). The overall accuracy is higher when the reference standard is based on ADC (ADC < 1.189×10^–3^ mm^2^/s) than when based on the short axis diameter (SAD) (SAD > 5.05 mm) [50]. A study by Giganti et al. proved that ADC value significantly differed according to local invasion, nodal involvement and the AJCC Cancer Staging Manual, 7th Edition TNM stage groups for GC, indicating that the ADC was potentially useful in the staging and risk stratification of GC patients [46]. Although Hasbahceci et al. [49] demonstrated that ADC value did not aid in distinguishing metastatic lymph nodes, this contrary conclusion was based on study of only 23 GC subjects and was not convincing. In addition, the ADC value correlates with the histological features, response to treatment and long-term prognosis [72–75]. The increased ADC signifies long-term survival [72]. Thus, the quantitative analysis measured by ADC value is a promising method for N-staging assessment in the future.

Although no wild heterogeneity was assessed by the I^2^ test among the selected studies of DWI, a wide variation in imaging techniques including preparations (gastric emptying, reduced peristole and filling-expansion of the stomach), instruments (field strength, pulse sequence, b value), procedures (breathholding, measuring method of ADC value) still existed [47–51]. These inconsistencies could inhibit the accuracy of DWI for staging [76, 77]. However, because of the limited number of included studies of DWI, no subgroup analyses were carried out to explore their impacts on the diagnostic performance of DWI. As a result, large-scale, high-quality trials are expected to standardize the preparations, parameters of instrument, procedures, and cutoff values of DWI for lymph node diagnosis.

Integrated PET/CT directly combines PET data on metabolic changes with highly detailed anatomic CT information, which help detect lesions earlier and provide more precise location information than CT or PET alone in numerous cancers [78]. Even though ^18^F-FDG PET/CT achieved inadequate sensitivity, it was not undertaken to evaluate lymph node metastasis in GC patients. On one hand, physiologic uptake was originally high in GC. Thus when primary tumor uptake was not dramatically increased, the detection of lymph node metastasis is difficult [79, 80]. On the other hand, most of the included studies only adopted the qualitative analysis by radiograph reading, without combining with the value of maximum standardized uptake (SUVmax) [21, 38–41, 52, 53, 55]. In our subgroup analysis, the quantitative analysis based on SUVmax displayed a much higher sensitivity than qualitative analysis (0.84 vs. 0.47), with the imaging analysis being regarded as a potential resource of heterogeneity in our meta-analysis [54]. In fact, a lack of unified criteria prevents confirmation of the diagnosis of lymph node metastasis and the cutoff values of SUVmax differing in quantitative analysis [54, 81, 82]. When coupled with the long scanning-acquisition time and expense, ^18^F-FDG PET/CT is not recommended as the first choice for clinically assessing lymph node staging in GC patients [38, 41, 80]. Finding another sensitive imaging agent and establishing the criteria for N-staging are proposed to improve the present situation of PET/CT [54, 83, 84].

The present meta-analysis has several limitations. First and foremost, no head-to-head comparison between MRI and ^18^F-FDG PET/CT were done in a single study, which might cause some bias in patient selection, or even adjustment. Second, the assessment of the two techniques for lymph node staging in some included studies were patient-based. A region-by-region or node-by-node comparison that could provide crucial information and a more accurate assessment was not performed in this study. Third, a wide variation in imaging techniques likely influenced the assessment of diagnostic accuracy of ^18^F-FDG PET/CT and DWI, which are potential resources of heterogeneity. Forth, no single reference standard strategy for the histopathologic analyses was applied, and a wide variation in histopathologic types of GC was found in all studies. This factor was not analyzed because it was too mixed to classify. Finally, potential publication bias was found in ^18^F-FDG PET/CT studies by use of Deeks’ funnel plot.

In conclusion, DWI achieved a higher sensitivity and equivalent specificity than ^18^F-FDG PET/CT in preoperative N-staging of GC patients. When the efficacy of MDCT was taken as a reference, DWI represented a complementary imaging technique and ^18^F-FDG PET/CT had limited usefulness in the preoperative assessment of N-staging. Therefore, large-scale randomized control trials are needed to confirm their clinical values and to establish reference standards for measurement, analysis, and cutoff values of lymph node diagnosis for both DWI and ^18^F-FDG PET/CT.

## MATERIALS AND METHODS

### Search strategy

A comprehensive computer-aided literature search of PubMed, Cochrane Library, and Embase databases was carried out to find relevant articles about DWI or PET/CT for N-staging in GC subjects (last update July 12th, 2017). We used a search algorithm based on a combination of the following parameters: (“DW-MRI” OR “diffusion-weighted magnetic resonance imaging”) OR (“FDG” OR “18F-FDG” OR “FDG-18F” OR “fluorodeoxyglucose” OR “PET/CT” OR “positron emission tomography/computed tomography”) AND (“stomach cancer” or “gastric cancer” or “stomach carcinoma” or “gastric carcinoma” or “GC”) AND (“lymph node metastasis” or “nodal metastases” or “lymphatic metastasis” or “lymph node involvement” or “nodal involvement” or “lymph node status” or “lymph node staging” or “N staging” or “TNM”).

### Inclusion and exclusion criteria

The inclusion criteria were as follows: (i) Studies investigating the diagnostic value of DWI or ^18^F-FDG PET/CT in distinguishing lymph node metastasis in GC were identified. (ii) Pathological analyses were used as the gold standard of diagnosis. (iii) The values of true positive, false positive, false negative, and true negative could be obtained or calculated in the original literature. (iv) Studies were based on a per-patient or per-lesion analysis. (v) For eligible studies with data published more than once, we only included the studies with the largest sample sizes.

The exclusion criteria were as follows: (i) Studies focused on DWI or ^18^F-FDG PET/CT in monitoring chemoradiotherapy response or prognosis rather than on lymph node diagnoses. (ii) Studies included subjects who received preoperative radiotherapy or chemotherapy, which might cause tumor down-staging. (iii) Articles were case reports, reviews, meeting abstracts, *in vitro* studies, or animal experiments for GC, or the studies had fewer than 20 samples. (iv) Studies had data errors in statistical analyses.

### Data extraction and quality assessment

Two reviewers (XZ and YL, respectively) independently reviewed titles and abstracts of the retrieved articles according to the above-mentioned selection criteria. Articles were excluded if clearly ineligible. Then the full-text versions of the selected articles were evaluated to determine their eligibility for inclusion. Finally, the above two reviewers cross-checked each independently selected study. Any controversy was resolved by consultation with a third author (BC). For each eligible study, the following information was extracted: first author, year of publication, country and ethnicity of the study subjects, study design, technique characteristics for DWI and ^18^F-FDG PET-CT, reference standard, and diagnostic criteria. The values of true-positive, false-positive, true-negative, and false-negative were also extracted. The methodological quality was assessed according to the revised tool of the Quality Assessment of Diagnostic Accuracy Studies (QUADAS-2) , which consists of 11 question items with responses “yes,” “no,” or “not available” [85]. Two reviewers (XZ and YL, respectively) independently extracted the relevant data and assessed the methodological quality from each included study. Any discrepancies were resolved by discussion.

### Statistical analysis

For patient-based analyses, we identified the pooled sensitivities and specificities of DWI and PET/CT, as well as their 95% CI using the weighted average method. The sROC curve was constructed for recruited studies and AUC was calculated to estimate the overall accuracy. Comparison between the two techniques was performed by use of the *Z* test, which could detect diagnostic differences between sensitivity, specificity, and AUC of the two imaging modalities. The following formula was used: *Z* = (VAL_1_−VAL_2_)/SORT (SE_1_^2^+SE_2_^2^). VAL indicated the means of sensitivity, specificity, and AUC, and SE was the standard error of corresponding variables.

To better understand the diagnostic performance of the two imaging techniques, we took the performance of MDCT for nodal staging of GC as a reference. The pooled estimates of sensitivity, specificity and AUC with 95% CIs was derived from Wang's meta-analysis, which was published in 2015 [43].

Heterogeneity among those eligible studies was assessed by the I^2^ test, with I^2^ > 50% suggesting mild heterogeneity among studies. When I^2^ index was higher than 50%, a random-effect model was used; otherwise, a fixed-model was used. If mild heterogeneity existed among those included studies, the potential sources of heterogeneity were identified by meta-regression and subgroup analyses. Threshold effect was an important additional source of variation in meta-analysis. To assess whether the threshold effect existed, the Spearman's correlation test was used.

Deeks’ funnel plots were to determine potential publication bias for DWI and ^18^F-FDG PET/CT in assessing preoperative N-staging of primary GC subjects. Stata 14.0 software was used to run all the statistical analyses. Values of *P* < 0.05 were considered statistically significant.
